# Programmed cell death: the battlefield between the host and alpha-herpesviruses and a potential avenue for cancer treatment

**DOI:** 10.18632/oncotarget.25694

**Published:** 2018-07-17

**Authors:** Chuankuo Zhao, Mingshu Wang, Anchun Cheng, Qiao Yang, Ying Wu, Dekang Zhu, Shun Chen, Mafeng Liu, XinXin Zhao, Renyong Jia, Kunfeng Sun, Xiaoyue Chen

**Affiliations:** ^1^ Institute of Preventive Veterinary Medicine, Sichuan Agricultural University, Wenjiang, Chengdu City 611130, Sichuan, P.R. China; ^2^ Key Laboratory of Animal Disease and Human Health of Sichuan Province, Sichuan Agricultural University, Wenjiang, Chengdu City 611130, Sichuan, P.R. China; ^3^ Avian Disease Research Center, College of Veterinary Medicine, Sichuan Agricultural University, Wenjiang, Chengdu City 611130, Sichuan, P.R. China

**Keywords:** alpha-herpesviruses, apoptosis, autophagy, necroptosis, cancer treatment

## Abstract

Programed cell death is an antiviral mechanism by which the host limits viral replication and protects uninfected cells. Many viruses encode proteins resistant to programed cell death to escape the host immune defenses, which indicates that programed cell death is more favorable for the host immune defense. Alpha-herpesviruses are pathogens that widely affect the health of humans and animals in different communities worldwide. Alpha-herpesviruses can induce apoptosis, autophagy and necroptosis through different molecular mechanisms. This review concisely illustrates the different pathways of apoptosis, autophagy, and necroptosis induced by alpha-herpesviruses. These pathways influence viral infection and replication and are a potential avenue for cancer treatment. This review will increase our understanding of the role of programed cell death in the host immune defense and provides new possibilities for cancer treatment.

## INTRODUCTION

Cell death can be either accidental or programed. Apoptosis was traditionally considered programed cell death (PCD) and is controlled by a series of cellular genes. Recently, many studies have shown that autophagy and necroptosis are also types of PCD. The molecular mechanism of apoptosis is clear; however, the molecular mechanisms of autophagy and necroptosis and their biological effects are hot spots of recent research. Viral clearance of immune cells through PCD is beneficial to virus infection, although many studies have proven that PCD is more beneficial to the host. As our understanding of innate immune defenses is mainly based on work with pathogens that evade these defenses, recognizing how PCD functions in response to infections is often difficult. Indeed, many discoveries of cell death functions are based on experiments with pathogens that have been genetically modified to remove their normal host-evasion strategies.

Alpha-herpesviruses belong to a subfamily of herpesviruses and are widely distributed in nature. The human alpha-herpesviruses include herpes simplex virus types 1 and 2 (HSV-1 and HSV-2) and varicella zoster virus (VZV). Moreover, several veterinary alpha-herpesviruses, including bovine herpesvirus-1 (BHV-1), caprine herpesvirus type 1 (CpHV-1), pseudorabies virus (PRV) and duck plague virus (DPV), have been identified [1–9]. Alpha-herpesviruses are double-stranded DNA viruses with genome sizes ranging from approximately 120 to 180 kb [10, 11]. These viruses consist of four major structural components: a central core where the viral DNA is located, a surrounding envelope composed of glycoproteins and of host cell membrane fragments, a tegument and a capsid [12–18]. Alpha-herpesviruses cause lytic and latent infections. During the lytic cycle, the transcription and replication of viral genome and the assembly of new capsids occur in the nucleus. Latent infection occurs when virions enter sensory neurons, where replication is infrequent because these cells do not divide. During latent infection, latency-associated transcripts are expressed and encode proteins that promote neuron and virus survival and that are needed for reactivation of the viral infection. Reactivation occurs when exposed to stress, heat, ultraviolet light, fever, hormonal changes, and nerve trauma [19–21].

## THE ROLES OF APOPTOSIS, AUTOPHAGY AND NECROPTOSIS IN ALPHA-HERPESVIRUS REPLICATION AND PATHOGENESIS

### The roles of apoptosis in alpha-herpesvirus replication and pathogenesis

**Figure 1:**
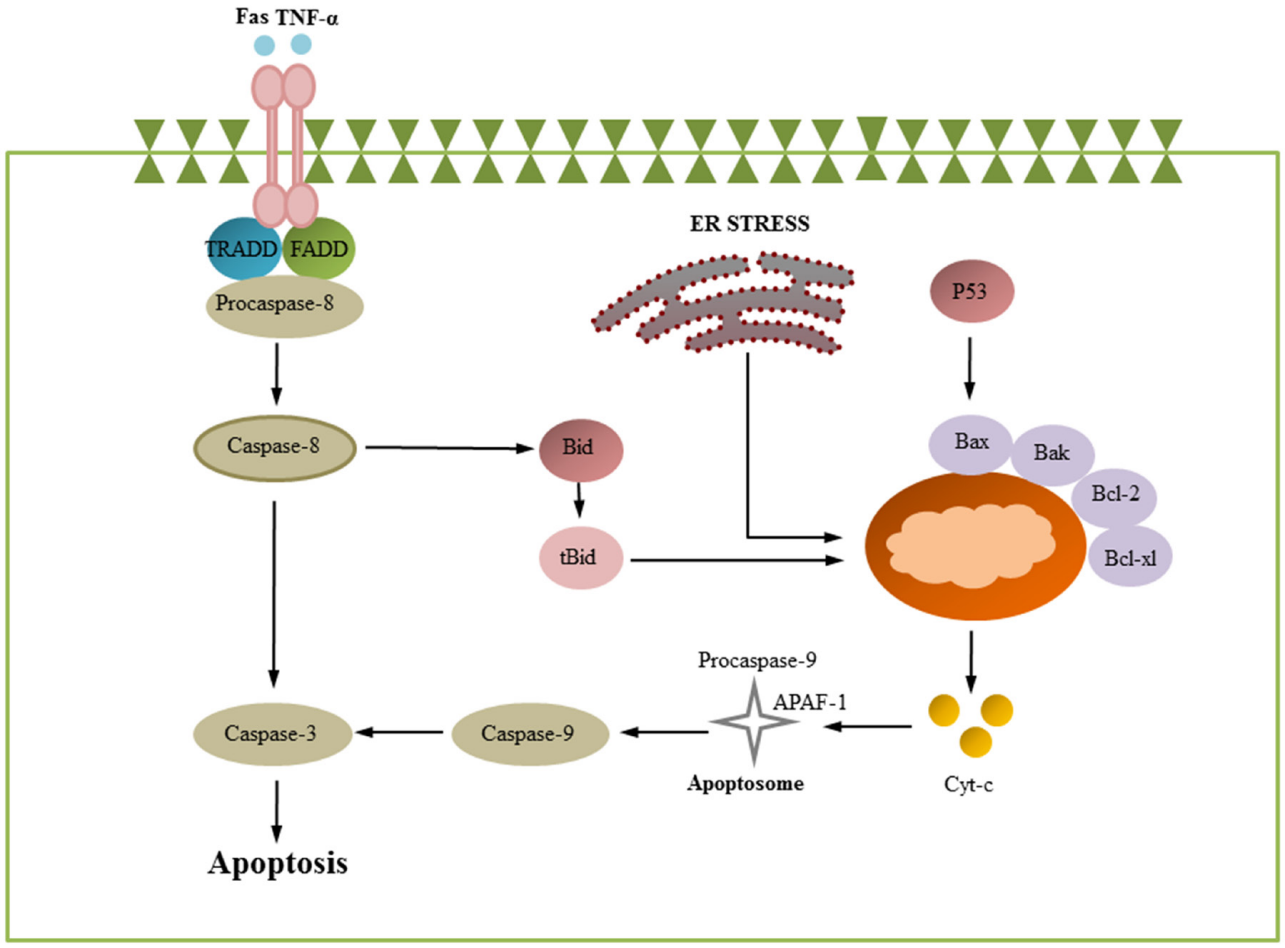
Apoptosis signaling pathways Apoptosis is initiated via two different routes: the mitochondrial pathway and death receptor pathway. The mitochondrial pathway is mainly regulated by bcl-2 protein family members. Pro-apoptotic and anti-apoptotic proteins are up- and downregulated, leading to decreased mitochondrial membrane potential and subsequent cytochrome *c* release from mitochondria. The released cytochrome *c* can later activate caspase 9, which in turn activates caspase-3. The extrinsic signals are initiated by cell death ligands (FasL, TNF and others) and activate FADD, which subsequently cleaves pro-caspase-8. Cleavage of pro-caspases-8 initiates the activation of caspase-8, which later can directly trigger effector caspases, including caspase-3 and caspase-7. (some inspiration came from these articles [1, 152, 74]).

Apoptosis is an important mechanism of the host immune defense because it can effectively clear the virus in infected cells. This process is mainly characterized by chromatin aggregation, cell concentration, and apoptosis body formation [22]. At present, apoptosis has been shown to be induced by two classical pathways: extrinsic and intrinsic apoptotic pathways. Caspases are cysteine proteases that are extremely important for intracellular apoptotic pathways. Caspase-9 and caspase-8 are involved in intrinsic and extrinsic apoptotic pathways, respectively [23, 24]. The extrinsic pathway is initiated by the binding of death ligands to interrelated receptors; the binding of death ligands (TNFR1, TNFR2, Fas, DR3, DR4, and DR5) and their receptors will recruit TRADD, FADD, and pro-caspase-8 to form the DISC complex and activate caspase-8. Subsequently, the downstream molecule caspase-3 is activated, and activated caspase-3 can cleave PARP to initiate cell apoptosis [23, 25, 26]. The intrinsic signaling pathways are known as mitochondria- and ER-initiated apoptosis [27, 28]. The mitochondrial apoptotic pathway is mainly controlled by the BCL-2 protein family. A series of Bcl-2 protein family members, including Bax, Bak, Bcl-2, Bcl-xl, Mcl-1, Bid, and Bim, can destroy the mitochondrial membrane potential (MMP). Cytochrome c (Cyt-c) is then released from the mitochondria into the cytoplasm and combines with pro-caspase-9 and Apaf-1 to form a complex to activate caspase-9, which subsequently activates the downstream molecules caspase-3 (Figure 1) [5, 29–31].

At present, many studies have shown that alpha-herpesviruses can induce apoptosis in various cells; however, alpha-herpesviruses can induce cell-specific apoptosis [2, 32, 33]. HSV-1 infects the liver and pituitary gland in mice, inducing apoptosis and leading to the formation of serious lesions. When the adrenal gland is infected by HSV-1, apoptosis is induced to inhibit HSV-1 replication. Similarly, in skin cells and immunocompromised corneal tissue, apoptosis is induced to inhibit HSV-1 replication. However, HSV-1 infection of *in vitro*-cultured epithelial cells and trigeminal nerve cells does not cause apoptosis [34–38]. Late-stage HSV-2-infected U937 cells can cause apoptosis and inhibit viral replication [39]. Moreover, HSV-2 infection can lead to T cell apoptosis to escape the host immune defense [40, 24]. Additionally, some animal herpesviruses also can induce apoptosis. BHV-1 infection of MDBK cells can induce apoptosis and inhibit BHV-1 replication [41, 32, 42–44], but it cannot induce the apoptosis of nerve cells. DPV can induce the apoptosis of uninfected lymphocytes *in vivo*, whereas apoptosis was not induced in infected lymphocytes [6]. DPV induced the apoptosis of peripheral uninfected lymphocytes o escape the host immune defense, while DPV infection of DEF cells can cause syncytium formation and apoptosis. Syncytium formation and apoptosis contribute to DPV pathogenesis [45]. PRV can induce apoptosis in many cells, and PRV infection of the trigeminal ganglion can induce or inhibit apoptosis. PRV pathogenesis and apoptosis have a certain relationship: when the virus causes increased rates of apoptosis, then host cells present serious lesions [46, 47]. Apoptosis can inhibit viral replication, but alpha-herpesviruses can encode many proteins to suppress or delay the progression of the virus itself (reviewed in (1)). Alpha-herpesviruses can control this subtle relationship between apoptosis and viral infection, promoting long-term survival of the viruses.

**Figure 2:**
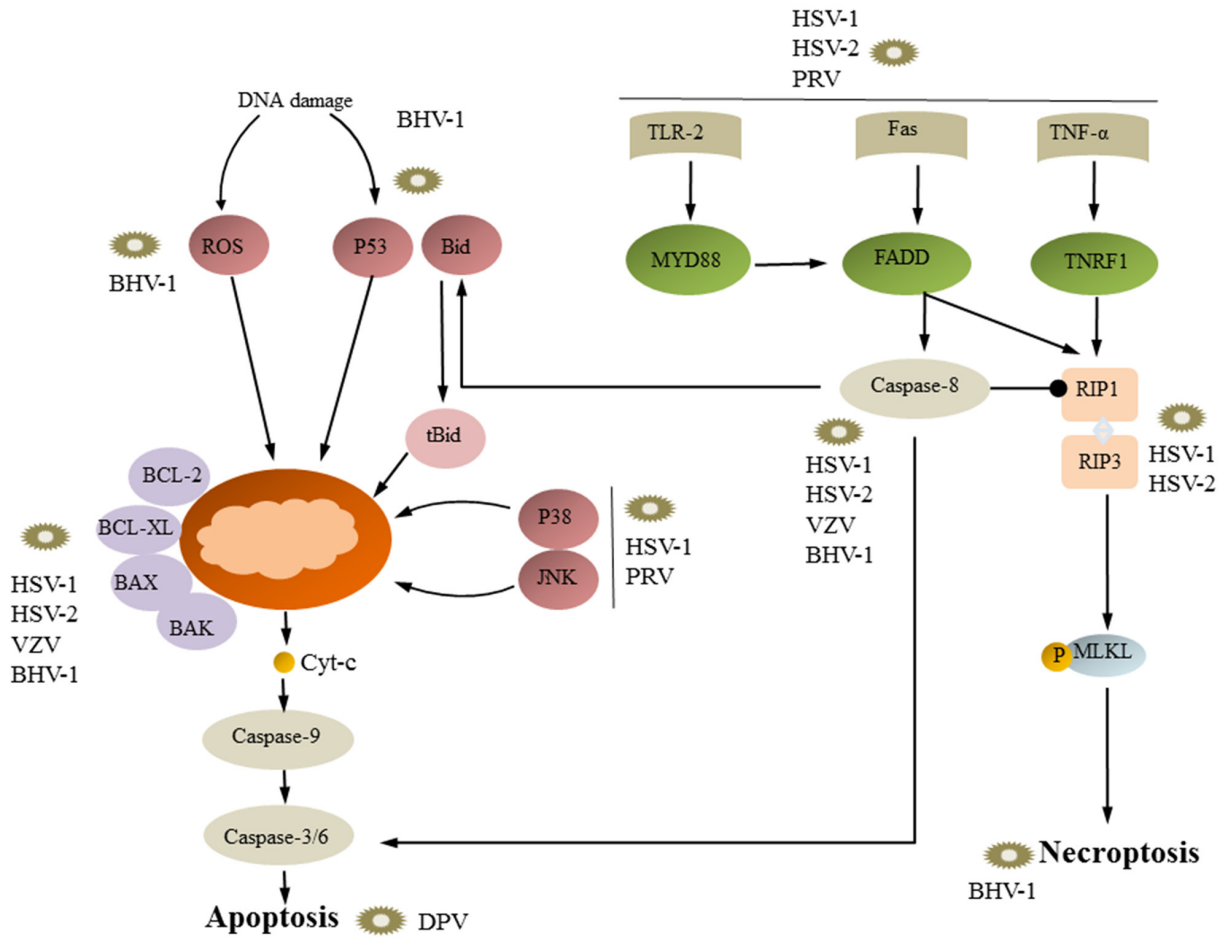
Necroptosis, apoptosis and viral replication Generally, viral infection can induce both intrinsic and extrinsic apoptotic pathways. Viruses such as HSV-1, HSV-2, and PRV initiate extrinsic signals through cell death ligands (e.g., FasL and TNF), causing caspases-8 activation, which then triggers caspase-3 and caspase-6. HSV-1, HSV-2, VZV, and BHV-1 directly trigger caspase-8; however, HSV-1, HSV-2, VZV, and BHV-1 target the BCL-2 protein, which initiates the mitochondrial pathway. BHV-1 affects the intrinsic pathway of apoptosis through stimulation of p53 and ROS. Once p53 is activated or intracellular ROS increases, mitochondria-dependent apoptosis can be activated. HSV-1 and -2 can inhibit apoptosis through inhibiting caspase-8 activity and RIP1 binding to RIP3. Then, RIP3 activates the downstream factor MLKL, leading to necroptosis.

The mechanisms of apoptosis induced by alpha-herpesviruses are complicated and can involve the intrinsic pathway and extrinsic pathway (Figure 2). The mitochondria play a dominant role in the intrinsic apoptotic pathway. BHV-1 infection of MDBK cells can reduce the MMP and promote apoptosis; after the MMP decreases, Cyt-c is released and activates caspase-9 [41]. The Bcl-2 protein family is important in the modulation of outer mitochondrial membrane integrity [29]. BHV-1 reduces the MMP by decreasing the level of the anti-apoptotic protein Bcl-2 and increasing the level of the pro-apoptotic protein Bax [41]. VZV infection of MeWo cells can reduce the mRNA expression of the anti-apoptotic protein Bcl-2, and after 48 hours and 60 hours, caspase-9 is activated [7, 48]. Similar to VZV, SVV can cause varicella in primates, and infected Vero cells show decreased mRNA levels of the anti-apoptotic protein Bcl-2 and subsequent activation of caspase-9 [30]. HSV-1 infection of human monocytes can active the pro-apoptotic proteins PUMA, Bax and Bak, which decrease the MMP and cause the release of Cyt-c from mitochondria to the cytoplasm, and caspase-9 is then activated by Cyt-c [31]. HSV-1 ICP27 is a multifunctional protein that plays a key role in apoptosis (66). ICP27 interacts with 14-3-3θ, which sequesters Bax to the cytoplasm. In addition, ICP27 promotes the translocation of Bax to the mitochondria by inhibiting the interaction between 14-3-3θ and Bax [49]. CpHV-1 induces apoptosis through the mitochondrial pathway. Mitochondria are also regulated by many BCL-2 family proteins; the levels of the pro-apoptotic proteins Bax and Bid increase, whereas the levels of the anti-apoptotic proteins Bcl-2 and Bcl-xl decrease [5, 50, 51]. The results from Vanden Oever indicate that HSV-2-induced apoptosis in T cells occurs via the intrinsic pathway, and caspase-9 is an important factor for HSV-2-induced apoptosis [24]. These data indicate that the intrinsic apoptotic pathway is involved in alpha-herpesvirus-induced apoptosis. However, ROS play an extremely important role in the early stages of apoptosis [52]. Mitochondria are both the target and source of ROS [53, 54]. When excess ROS are generated, they induce MMP depolarization and Cyt-c release, which triggers caspase activation [55]. ROS generation can also induce DNA damage, which then promotes apoptosis [56]. BHV-1 infection increases ROS production, which depends on viral entry, viral protein expression or DNA replication [57]. Excessive ROS induce MMP depolarization, and then Cyt-c is released from mitochondria and activates caspase-9. HSV-1 ICP27 is a multifunctional protein that plays a key role in apoptosis. Kim et al. demonstrated that ICP27 induces apoptosis through ROS generation and that ROS induce apoptosis by increasing AP-1 activity and Bax expression and reducing Bcl-2 expression and activated cell cycle checkpoints in 3-3 cells [58].

However, many studies have examined the alpha-herpesvirus-induced extrinsic apoptotic pathway. Xu et al. suggested that BHV-1 can increase the expression of Fas, Fasl, caspase, caspase-3, and caspase-8; however, the BHV-1-induced extrinsic pathway is not isolated and can connect with the mitochondrial apoptotic pathway. Activated caspase-8 can cleave Bid, and the cleaved form, tBid, translocates to the mitochondria, where it promotes Cyt-c release [41]. CpHV-1 causes apoptosis in mitogen-stimulated and unstimulated caprine peripheral blood mononuclear cells (PBMCs). CpHV-1 can also active caspase-8, caspase-9 and caspase-3, and Bid is cleaved, forming tbid, which suggests that “cross-talk” between the death receptor pathway and the mitochondrial pathway occurs in CpHV-1-induced apoptosis *in vitro* [51]. PRV infection increases TNF-alpha transcription, translation and secretion, as well as TNF-alpha receptor expression [59]. These data indicate that the extrinsic apoptotic pathway is involved in alpha-herpesvirus-induced apoptosis.

In addition, some other factors are involved in the apoptosis induced by alpha-herpesvirus. p53 is a tumor suppressor gene whose biological function is to monitor the integrity of DNA in the G phase [60, 61]. If DNA is damaged, p53 inhibits cell proliferation until DNA repair is complete [62], whereas if the DNA cannot be repaired, apoptosis is induced [60]. BHV-1 induces apoptosis in the G0/G1 phase of the cell cycle by increasing the protein level of p53 [63]. Another study demonstrated that CpHV-1 causes nerve cell apoptosis by significantly increasing p53 protein levels and p53 phosphorylation levels [5]. Several different subfamilies of MAPKs have been identified in mammalian cells [64]. These MAPK family members include extracellular signal-regulated kinases (ERKs), including ERK1 and ERK2; JNKs/SAPKs, including p54 SAPK (SAPKα/β, JNK2) and p45 SAPK (SAPKγ, JNK1); and p38 MAP kinases. JNK/SAPK and p38 MAPK have been shown to phosphorylate a number of transcription factors, such as c-Jun and ATF-2. c-Jun is phosphorylated specifically by JNK/SAPK, and ATF-2 can be phosphorylated by both JNK/SAPK and p38 MAPK. All these pathways can induce apoptosis [65]. HSV-1 infection causes apoptosis in cultured cells and causes *in vivo* activation of p38 and c-Jun N-terminal kinase/stress-activated protein kinases [66, 34] HSV-1 ICP27 activates the p38 and JNK signaling pathways, leading to host cell apoptosis [67]. Yeh et al. showed that PRV infection of host cells can activate p38 MAPK and JNK/SAPK signaling [59]. In addition, HSV-1 infection of cells can active TLR 2, which is important for host innate immune; TLR2 participates in HSV-1-induced apoptosis [68, 69].

### Autophagy and alpha-herpesvirus replication

**Figure 3:**
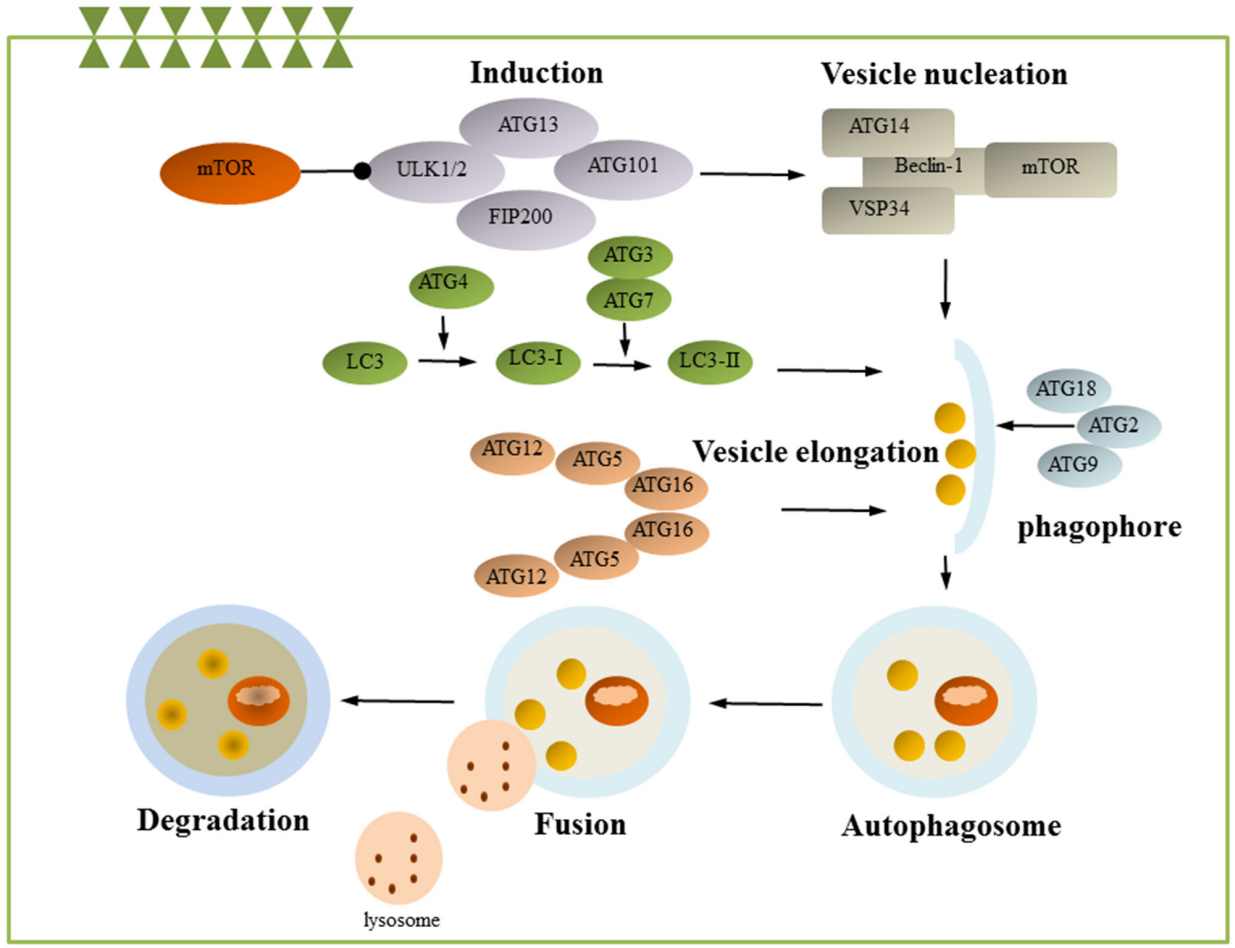
Autophagy Autophagy is a process by which damaged or unnecessary cellular compartments are degraded and recycled. This process has several tightly regulated steps, including induction, nucleation, expansion, completion, fusion and degradation. mTOR is known as the key regulator of autophagy induction and can be suppressed by ULK1, leading to VPS34-Beclin 1-class III PI3-kinase complex activation. Several different membrane pools contribute to the formation of the phagophore. The Atg proteins (Atg2, Atg9, and Atg18) are essential for phagophore formation. The ATG and LC3 conjugation system also contributes to autophagosome membrane formation and elongation. The autophagolysosome is then formed by fusion of the autophagosome with a lysosome to degrade and reuse the compounds. ATG, autophagy-related genes; mTOR, mammalian target of rapamycin. (some inspiration came from these articles [153, 74, 81, 70]).

Autophagy is an evolutionarily conserved catabolic process in which intracellular membrane structures package protein complexes and organelles to degrade and renew these cytoplasmic components [70]. The autophagosome then fuses with the lysosome, and its contents are degraded by lysosomal enzymes. Autophagosomes can also selectively target/engulf ubiquitinated cargo (selective autophagy), mitochondria (mitophagy), or pathogens (xenophagy) [71–73]. In mammals, temperature changes, and in particular heat shock, can stimulate autophagy [74, 75]. More than 30 kinds of ATG are involved in autophagy induction and can be divided into the following complexes according to their functions: the ULK complex, PI3K complex, ATG12 complex, and ATG8 complex [76, 77]. The ULK complex, which is composed of ULK1, ULK2, ATG13, ATG101, and the focal adhesion kinase family-interacting protein of 200 kDa (FIP200), is crucial for autophagy induction [78]. Mammalian target of rapamycin complex 1 (mTORC1) binds to and inactivates ULK1 and ULK2. The dissociation of mTORC1 from the ULK complex leads to ULK1/2 activation and the subsequent phosphorylation of FIP200 and ATG13, which initiates phagophore formation [79]. PI3K complexes, which play an important role in the initiation of vesicle nucleation, include P150, PI3 class III, Beclin-1 and ATG14 and are regulated by Bcl-2, Bcl-xL, and Ambra 1 [77]. ATG12 complexes and ATG8 complexes play an important role in autophagosome formation. In the Atg12 conjugation system, Atg12 is activated by Atg7 and Atg10 and conjugated to Atg5, which promotes the formation of the autophagy precursor. Atg5 interacts further with autophagy-related 16-like1 (ATG16L1) to form the ATG16L1-ATG5-ATG12 complex [80]. LC3, the mammalian ortholog of Atg8, is cleaved by Atg4 to form LC3-I. LC-I then bonds with the E1-like enzyme Atg7 and is transferred to the E2-like enzyme Atg3. Finally, LC-I is conjugated to PE to form LC3-II, which is necessary during the elongation step of autophagy (Figure 3) [81].

**Figure 4:**
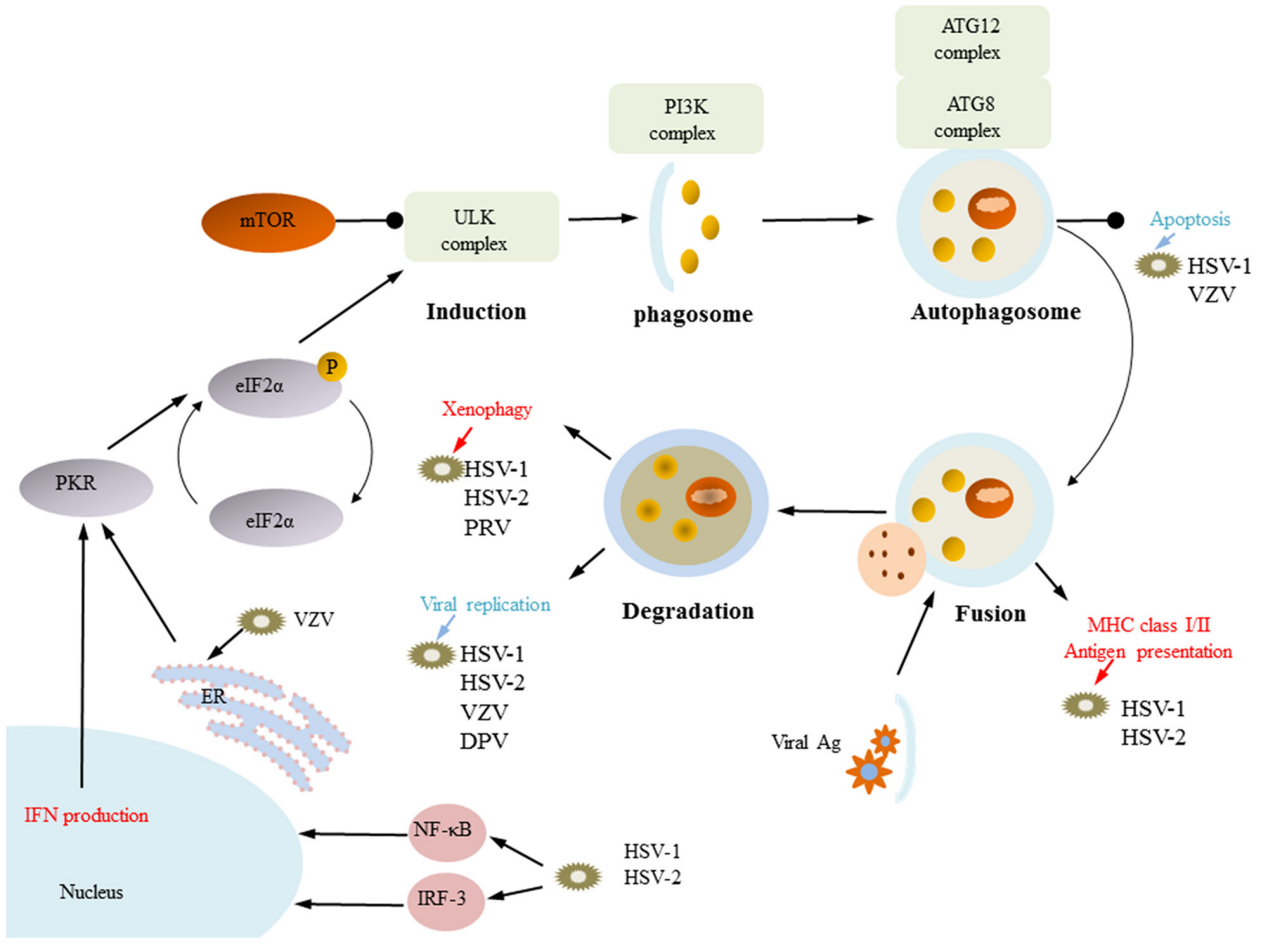
Functions of autophagy HSV-1, HSV-2, VZV, EHV-1, PRV and DPV have been found to induce autophagy by different molecular mechanisms. HSV-1, HSV-2, EHV-1, and PRV use xenophagy to remove the virus from infected cells, and they can use autophagy combined with innate immune and acquired immune defense to promote viral infection. HSV-1 and HSV-2 infection of cells can produce IFN-induced autophagy, and autophagy participates in MHC-I/MHC-II antigen delivery. For VZV, DPV, HSV-1 and HSV-2, induction of autophagosome formation is also important for viral replication. VZV induces autophagy through ER stress. (some inspiration came from these articles [154, 74, 80]).

The alpha-herpesviruses HSV-1, HSV-2, VZV, EHV-1, PRV and DPV have been found to induce autophagy by different molecular mechanisms. Autophagy can engulf HSV-1, HSV-2, and EHV-1-infected cells and can work in combination with innate immunity and acquired immune defense virus infection. However, a subsequent study also found that HSV-1 and HSV-2 use autophagy to complete their own replication. In addition, VZV and DPV can cause complete autophagy, but they use only autophagy to complete their own protein synthesis and replication (Figure 4).

#### Autophagy inhibits alpha-herpesvirus replication

Autophagy is an important host immune defense mechanism. One type of autophagy that can engulf infected cells to kill the virus is called xenophagy [82]. In the early stages, both PRV and UV-inactivated PRV infected Vero cells can induce autophagy, suggesting that viral gene expression is not required to induce autophagy. The inhibition of autophagy by rapamycin contributes to PRV replication, suggesting that PRV-induced autophagy can inhibit viral replication. The PRV US3 protein is involved not only in the regulation of apoptosis but also in the inhibition of apoptosis via the AKT/mTOR pathway; thus, PRV US3 should be further studied to determine the relationship between apoptosis and autophagy [83, 84]. HSV-1-infected cells activate EIF2AK2, which is the host defense molecule. EIF2AK2 can phosphorylate EIF2S1/eIF2α and promote autophagy and host shut-off protein synthesis; inhibition of EIF2S1/eIF2α phosphorylation can inhibit the host shut-off protein synthesis and viral replication [85, 86]. HSV-1 ICP34.5 is able to recruit the host-phosphorylated protein PPP1CA/PP1α, which can dephosphorylate EIF2S1. Moreover, HSV-1 ICP34.5 can bind BECN1 to inhibit BECN1-induced autophagy [87–89]. HSV-1 ICP34.5-deleted mutants show increased autophagy compared with wild-type HSV-1-infected cells, and HSV-1 ICP34.5-deleted mutants can be degraded by autophagy, which suggests that autophagy can inhibit HSV-1 infection [90, 91]. However, Alexander et al. found that HSV-1-induced autophagy has no effect on viral replication, that HSV-1 ICP34.5-deleted mutants cause autophagy, and that inhibition of HSV-1 ICP34.5-deleted mutant-induced autophagy has no effect on viral replication [92]. The early stages of EHV-1 infection of cultured murine neurons causes autophagy but has no effect on EHV-1 replication [93]. This seemingly contradictory outcome has a reasonable explanation: on the one hand, other forms of autophagy in addition to xenophagy may be involved in immune regulation, whereas on the other hand, other factors initiated by innate immune and acquired immunity may be involved in immune regulation.

Many studies have shown that in addition to xenophagy removing the virus, autophagy can correlate with innate immune and acquired immunity defense [94–96]. However, these hypotheses have limitations because they are based on *in vitro* cell culture. The innate immune response is an important means of resistance to viral infection, and viruses encode many genes to avoid innate immunity [97]. The host cells recognize viruses through the pattern receptor and subsequently activate the important transcription factor IRF/NF-ΚB, which regulates ISG to regulate IFN production [98, 99]. Alpha-herpesviruses can activate the IFN signaling pathway to induce an antiviral effect, and IFN can cause antiviral effects in a variety of ways, including autophagy. HSV-1 can stimulate IFN-γ to cause autophagy, which can inhibit viral replication. Katzenell et al. also found that HSV-1 induced the formation of new autophagosomes greater than 4 µm via the IFN signaling pathway, and ISG5 molecules were found in these new autophagosomes [100–102]. Alpha-herpesvirus-induced autophagy participates in the antigen presentation process, which activates acquired immunity. HSV-1 ICP34.5 can bind to Beclin-1 via the BBD motif to inhibit autophagy. Mice infected with the HSV-1 ICP34.5 BBD-deleted mutant show stimulated CD4+ T cell responses and clear the virus more efficiently than mice infected with the wild-type virus. In addition, autophagy also facilitates the CD8+ T cell response. Inhibition of the host translation shutoff response by HSV-1 triggers nuclear envelope-derived autophagy. This unique autophagosome is able to ‘cross-present’ endogenous HSV-1 antigens within the MHC-I pathway, enabling responses from CD8^+^ T cells. Here, we provide evidence that the infection of macrophages with HSV-1 triggers a vacuolar response that increases the presentation of the HSV-1 peptide glycoprotein B (gB) to CD8+ T cells on MHC class I molecules. This vacuolar response, which is linked to autophagy, can be modulated by various cytokines and stress conditions. In line with these findings, mutant HSV-1, which is unable to suppress autophagy, causes increased proliferation of CD8+ T cells responding to virally infected cells compared to the outcome with the wild-type virus. Above all, several studies have shown that autophagy participates in the antigen presentation process and may serve as a target for the host to boost adaptive immunity against HSV-1 infection [103–106].

#### Autophagy promotes alpha-herpesvirus replication

Autophagy can not only stabilize the intracellular environment but also combine with innate immune and acquired immune defenses against virus infection. However, many studies have shown that alpha-herpesvirus can take advantage of autophagy to promote the virus life cycle; this is called “pro-virus autophagy”. Early-stage HSV-1 infection of THP-1 cells causes autophagy independent of viral gene expression. Then, HSV-1 promotes autophagosome formation via the MYD88 signaling pathway; drug inhibitors or siRNA inhibition of autophagosome production can prevent viral replication [107]. HSV-2 infection of cells can inhibit autophagy but still cause basal autophagy, and Bafilomycin A1 (BFN) or ATG5 knockout can inhibit basal autophagy, which seriously interferes with viral replication [108]. VZV differs from the other two human herpesviruses because it lacks homologs of both HSV-1/HSV-2 ICP34.5 and US11, which are inhibitory autophagy proteins [109, 110]. VZV, LC3 and the endoplasmic circulatory marker Rab 11 are co-localized together, and Rab 11 regulates endoplasmic membrane fusion to aid autophagic membrane formation via ATG9 and ATG16L1 dependency. Moreover, VZV infection of cells can induce ER stress, and then VZV uses ER stress to induce autophagy. This autophagy has an important role in viral glycoprotein synthesis and replication; drug inhibitors or siRNA silencing of ATG5 inhibits VZV-induced autophagy, which seriously interferes with viral glycoprotein synthesis and replication [111–113]. However, avian herpesvirus DPV infection increases the expression of the autophagy marker protein LC3-I. LC3-II reduced P62/SQSTM1 protein expression after DEF cells were infected, indicating that DPV infection of cells can cause autophagic flux. siRNA silencing of an autophagy-related gene inhibited autophagy and DPV replication; however, the use of wortmannin, which inhibits autophagy in cells infected with DPV, decreased DPV replication [114].

Many studies have shown that regardless of the molecular mechanism or biological function, there is some relationship between apoptosis and autophagy. HSV-1 is able to prolong cell survival by autophagic resistance to apoptosis via the non-dependent mTOR pathway [115]. In addition, HSV-1 and HSV-2 infection of SIRC cells inhibited the occurrence of autophagy to increase the number of apoptotic cells, indicating that autophagy could resist the occurrence of apoptosis [116](121). Autophagy formation was detected in VZV-infected MRC-5 cells before 72 hours of infection, and a high percentage of apoptosis occurred after 72 hours. Autophagy may be able to resist the apoptosis that occurs in the early stage of infection [48, 109].

### Necroptosis and alpha-herpesvirus

**Figure 5:**
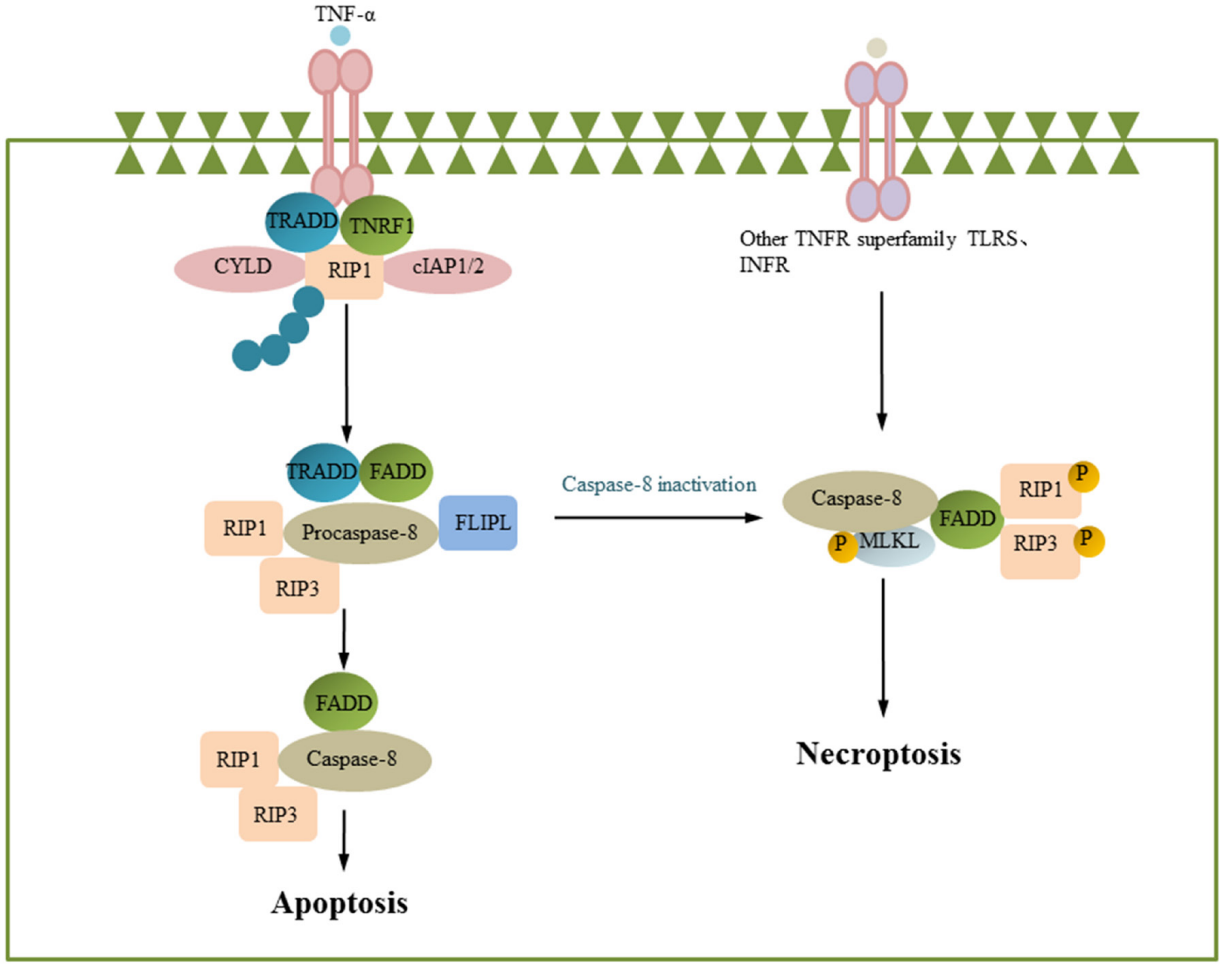
Necroptosis Engagement of the death receptor TNFR1 triggers the assembly of the membrane-proximal complex I composed of TNFR1, TRADD, RIP1, cIAP1/2 and TRAF2/5. Depending on the context, complex I can also undergo endocytosis, resulting in dissociation of TNFR1, deubiquitination of RIP1 by CYLD and recruitment of FADD and pro-caspase-8 to form complex II. Within complex II a, pro-caspase-8 is activated and can promote apoptosis by activating the caspase cascade. Caspase-8 can also cleave and inactivate RIP1 and RIP3, inhibiting necrosis. However, if caspase-8 is inhibited, RIP1 recruits RIP3 to complex II b. Then, RIP3 activates the downstream factor MLKL, leading to necroptosis. (some inspiration came from these articles [79, 81, 77]).

Necrosis is generally considered passive cell death caused by factors including infection and toxins; however, recent studies have shown that necrosis is also a type of programed death known as programed necrosis or necroptosis [117]. Necroptosis is usually triggered by TNF receptor family members, T cell receptors (TCRs), Toll-like receptor (TLRs), interferon receptor (IFNRs), or ER stress [118–121]. The RIP protein family members RIPK1 and RIPK3 play an important role in necroptosis and are related to multiple necroptotic pathways [119]. TNF receptor can recruit TRADD, TRAF2, RIP1, CYCLD, and cIAP1/2 to form complex I. cIAP1/2 can ubiquitinate RIP1, preventing it from forming complex II a (caspase-8, FADD and RIP1) and complex II b (Caspase-8, FADD, RIP1, RIP3, and MLKL). If CYCLD relieves RIP1 ubiquitination, complex II a formation is promoted, and then, caspase-8 activates the cleavage of RIP1 and RIP3. If caspase-8 is not activated, RIP1 and RIP3 can form RIP1-RIP3 complexes through their respective RHIM domains [122, 123]. Then, RIP3 activates the downstream factor MLKL/PGAM5; MLKL is cleaved and transferred to the cytoplasmic membrane, causing necroptosis; and PGAM5 can recruit and activate DRP1, reducing the MMP and leading to necroptosis [124–127]. In addition, TLR can induce necrotic apoptosis by combining Toll/IL-1 RHIM with RIP3 to form the TRIF/TICAM-RIP3 complex. This necroptotic pathway relies only on RIP3. In murine cytomegalovirus (MCMV)-infected cells, intracellular DAI can combine with RIP3 to form the DAI-RIP3 complex and induce necroptosis [128]. Thus, RIP3 and MLKL are indispensable molecules that cause necroptosis (Figure 5).

Necroptosis is an immune defense mechanism in which caspase-8 activity is inhibited and apoptosis cannot occur. Current studies have shown that human herpesviruses HSV-1 and HSV-2 can cause necroptosis of murine cells and human cells and that veterinary herpesviruses BHV-1 and BHV-5 can cause necroptosis of nerve cells [129, 130]. HSV R1 protein (HSV-1 ICP6 and HSV-2 ICP10) plays an important role in the regulation of apoptosis and necroptosis. The R1 protein has an N-terminal RHIM region and a C-terminal RNR (ribonucleotide reductase domain) region. This protein can inhibit apoptosis through the RNR region binding to caspase-8; this binding inhibits caspase-8 activity. The R1 protein can bind to RIP1/RIP3, which are associated with necroptosis, through the RHIM region [130–133]. HSV-1 ICP6-deleted mutants and HSV-1 ICP6 RHIM-deleted mutants resulted in necroptosis of infected cells, and the number of necroptotic cells induced by HSV-1 was twice the number of necroptotic cells induced by HSV-1 ICP6-deleted mutants and HSV-1 ICP6 RHIM-deleted mutants, indicating that in addition to ICP6, other HSV-1 proteins can promote necroptosis [123]. HSV-1 ICP6 can induce RIP3/MLKL-mediated necroptosis and does not depend on TNFR, TLR3, and DAI in infected mouse cells [134]. In contrast, HSV-1 ICP6 is able to inhibit TNF-induced necroptosis when human cells are infected, which may be due to differences in human and murine RIHM, resulting in the induction and suppression of necroptosis in different species (Figure 2) [135].

## THE ROLES OF APOPTOSIS, AUTOPHAGY AND NECROPTOSIS IN ALPHA-HERPESVIRUS TREATMENT OF CANCER

Viruses provide a unique platform for the treatment of cancer. The past two decades have witnessed increased interest in oncolytic viruses (OVs) as cancer therapeutics. OVs are naturally occurring or engineered viruses that selectively infect and replicate in cancer cells and cancer-associated endothelial cells, triggering direct oncolysis [136]. Apoptosis, necrosis/necroptosis, pyroptosis, autophagy and host immune system are important mechanisms by which OVs treat tumors [136, 137]. Interestingly, HSV-1, HSV-2, BHV-1, and EHV-1 are candidates for OVs. Oncolytic HSV-1 has been developed based on deletion of the ICP6, ICP34.5 and ICP47 genes and early expression of US11 to increase tumor-selective replication and increase antitumor immunity [138–140]. Mutated HSV-1 (NV1066) with ICP34.5 deleted is used to infect human gastric cancer cells and induce apoptosis. Interestingly, a significant percentage of uninfected cells also proceeded to apoptosis, which inhibited viral replication [141]. G47Δ is a mutant that contains three mutations: ICP34.5, ICP6, and ICP47. G47Δ-infected MCF-7/TAM-R cells present induced cell cycle arrest in G2/M phase and mass apoptosis [142]. G47Δ also increased the antitumor ability of paclitaxel by inducing mitotic arrest and apoptosis [143]. T-Vec is a double-mutated HSV-1 with deletions in the ICP34.5 and ICP47 genes, and the insertion of the human granulocyte-macrophage colony-stimulating factor (GM-CSF) gene into the deleted ICP34.5 can cause human tumor cell apoptosis [144]. The N-terminus of the HSV-2 ICP10 gene product contains a well-defined serine/threonine protein kinase (PK) domain, which can activate the Ras/MEK/MAPK mitogenic pathway and thus facilitate efficient HSV-2 replication [145]. Because the Ras signaling pathway is a key regulator of normal cell growth and malignant transformation, it is aberrantly activated in many human tumors [146]. HSV-2 ICP10 PK-deleted mutants have robust melanoma oncolytic activity in culture and in animal models (xenografts) through the simultaneous activation of multiple nonredundant PCD pathways. These pathways include the activation of distinct proteases and are associated with upregulation of the autophagy protein Beclin-1 and pro-apoptotic caspase-3, caspase-7, and H11/HspB8 [147–149]. BHV-1 is a species-specific herpesvirus closely related to HSV-1. Although BHV-1 does not efficiently replicate in and affect the cellular viability of normal human cells, it is capable of infecting and killing various immortalized and transformed human cell types. Furthermore, although some cross-reactivity between BHV-1 and HSV-1 exists, the majority of human antibody or serum samples tested failed to neutralize BHV-1 despite possessing HSV-1 neutralizing capacity [150]. Moreover, BHV-1 infection of neuronal- and glial-derived tumor cell cultures can induce ROS production, mitochondrial membrane dysfunction, apoptotic cell death and necroptotic cell death [129]. Another animal herpesvirus, EHV-1, productively infects human glioblastoma cell lines and significantly induces apoptosis, and the degree of infection is positively correlated with glioma cell death [151].

## PERSPECTIVE

This article reviews the mechanisms of apoptosis, autophagy and necroptosis induced by alpha-herpesviruses and their effects on viral replication and infection. However, many questions have not been resolved: How are apoptosis, autophagy and necroptosis related to each other? How do alpha-herpesviruses promote the survival of cells by autophagy, which inhibits apoptosis? When alpha-herpesviruses inhibit apoptosis, how does the host cell become necroptosis resistant via the virus. In addition, how do host cells resisting alpha-herpesvirus replicate at the same time through autophagy and apoptosis? Is there a link between autophagy and necroptosis that is resistant to alpha-herpesvirus infection?

Many studies have shown that HSV-1, HSV-2, EHV-1 and BHV-1 can be used as OVs in the treatment of tumors through apoptosis, autophagy and necroptosis. The mechanisms of PCD induced by alpha-herpesviruses will aid in the development of drugs to treat tumors. Moreover, the genomes of alpha-herpesviruses encode many genes related to inhibition of apoptosis, autophagy, and necroptosis to promote viral replication and infection. To understand the relationship between viruses and PCD, future studies to develop drugs to prevent alpha-herpesvirus infection should target the mechanisms of apoptosis, autophagy and necroptosis.
